# Supplementary material for: Effect of the anodization voltage on the pore-widening rate of nanoporous anodic alumina
Source: Nanoscale Res Lett. 2012 Aug 23;7(1):474. doi: 10.1186/1556-276X-7-474 (PMC3460793; doi:10.1186/1556-276X-7-474)
Supplement: Additional file 1 — Additional documentation: Contains Tables S1 and S2, and Figures S1 and S2. [file 1556-276X-7-474-S1.doc]

ADDITIONAL DOCUMENTATION

Table S1: List of the produced samples with the fabrication (anodization and pore widening) parameters.

| **Sample name** | **Porous Anodic Alumina Growth Conditions** | | | | |
| --- | --- | --- | --- | --- | --- |
|  | **Anodization Voltage (V) 2nd step** | **Anodization Time step2 (min)** | **Temperature 2^nd^ step T^0^ (C)** | **Total charge (Coulomb)** | **Pore Widening time(min)** |
| **110928.Al1** | **20** | **10** | **5** | **0,8** | **0** |
| **110928.Al2** |  | **15** | **6** | **1,1** | **3** |
| **110928.Al3** |  | **20** | **6** | **1,6** | **6** |
| **110928.Al4** |  | **25** | **5** | **1,8** | **9** |
| **110929.Al1** | **30** | **10** | **6** | **1.2** | **0** |
| **110929.Al2** |  | **15** | **6** | **1,9** | **3** |
| **110929.Al3** |  | **20** | **7** | **2.8** | **6** |
| **110929.Al4** |  | **25** | **5** | **3.5** | **9** |
| **111005.Al1** | **40** | **10** | **5** | **2.6** | **0** |
| **111005.Al2** |  | **15** | **5** | **4.2** | **3** |
| **111005.Al3** |  | **20** | **6** | **5.2** | **6** |
| **111005.Al4** |  | **25** | **5** | **8.4** | **9** |
| **111006.Al1** | **50** | **10** | **5** | **4,9** | **0** |
| **111006.Al2** |  | **15** | **5** | **7,3** | **3** |
| **111006.Al3** |  | **20** | **5** | **9,6** | **6** |
| **111006.Al4** |  | **25** | **5** | **11,2** | **9** |

Table S2: porosity and thickness obtained from the fitting of ellipsometric measurements.

| **Sample name** | **Anodization voltage (V)** | **Porosity (%)** | **Thickness (nm)** |
| --- | --- | --- | --- |
| **110928.Al1** | **20** | **28** | **201** |
| **110928.Al2** |  | **42** | **285** |
| **110928.Al3** |  | **59** | **396** |
| **110928.Al4** |  | **69** | **455** |
| **110929.Al2** | **30** | **39** | **520** |
| **110929.Al3** |  | **46** | **753** |
| **110929.Al4** |  | **56** | **958** |
| **111005.Al1** | **40** | **25** | **711** |
| **111005.Al2** |  | **31** | **1098** |
| **111005.Al3** |  | **39** | **1398** |
| **111005.Al4** |  | **44** | **1697** |
| **111006.Al1** | **50** | **23** | **1349** |
| **111006.Al2** |  | **30** | **1989** |
| **111006.Al3** |  | **33** | **2620** |
| **111006.Al4** |  | **40** | **3100** |

Figure S1. Current-time transients of all the produced samples. a) Samples produced with 10 minutes of anodization. b) Samples produced with 15 minutes of anodization. c) Samples produced with 20 minutes of anodization. d) Samples produced with 25 minutes of anodization.

Figure S2. Cross-section ESEM pictures of some of the samples and estimation of thickness.


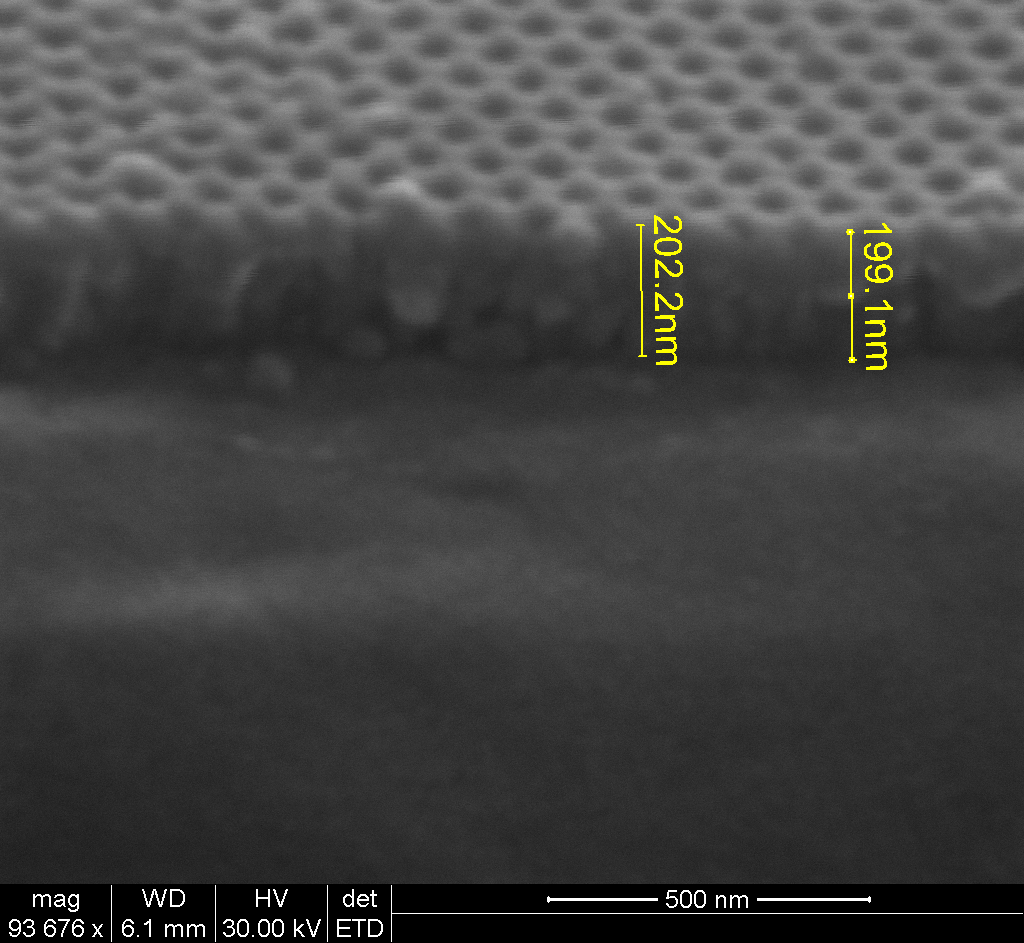

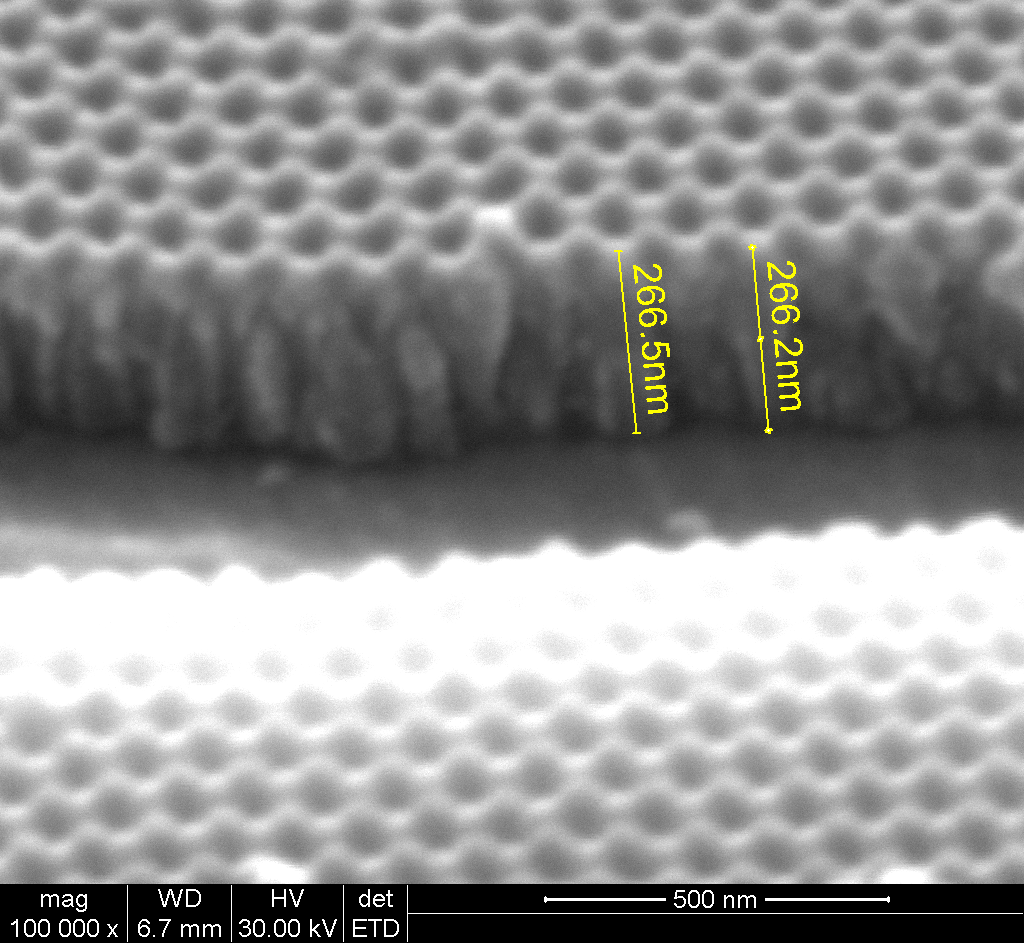


110928-Al1 (20V)

110929-Al1 (30V)


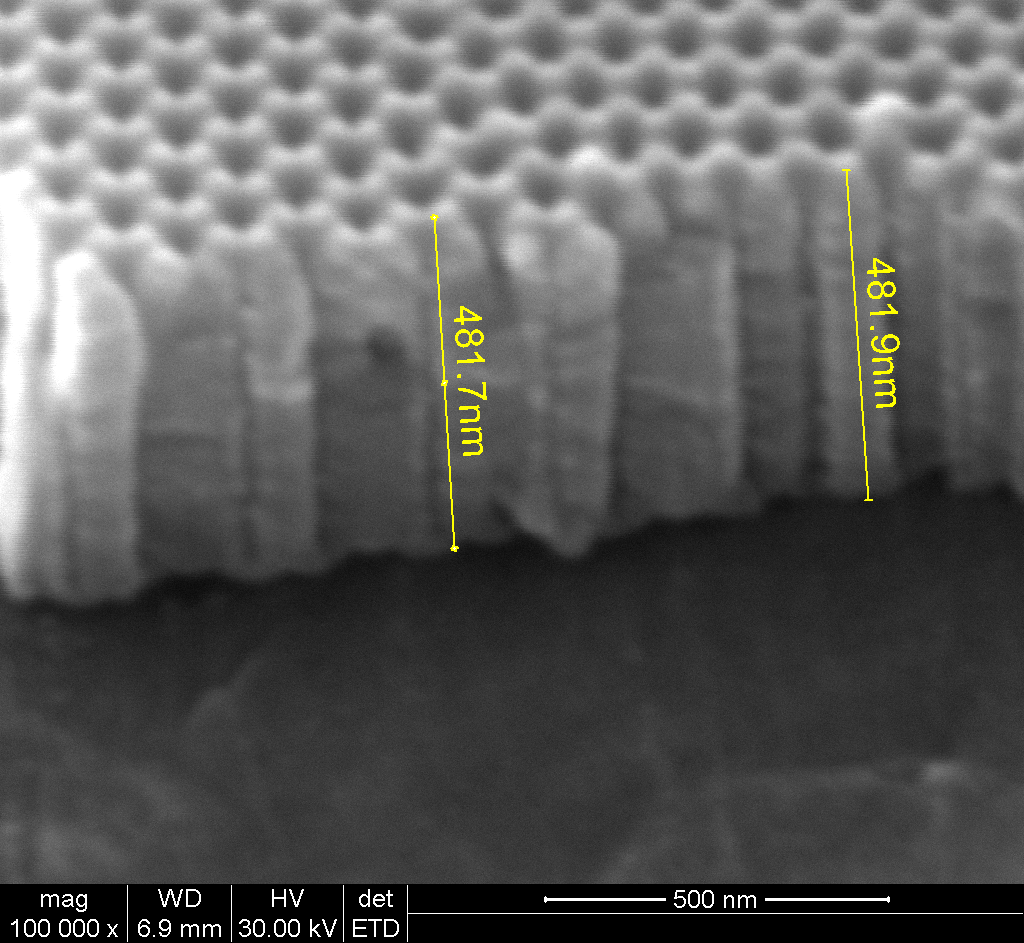

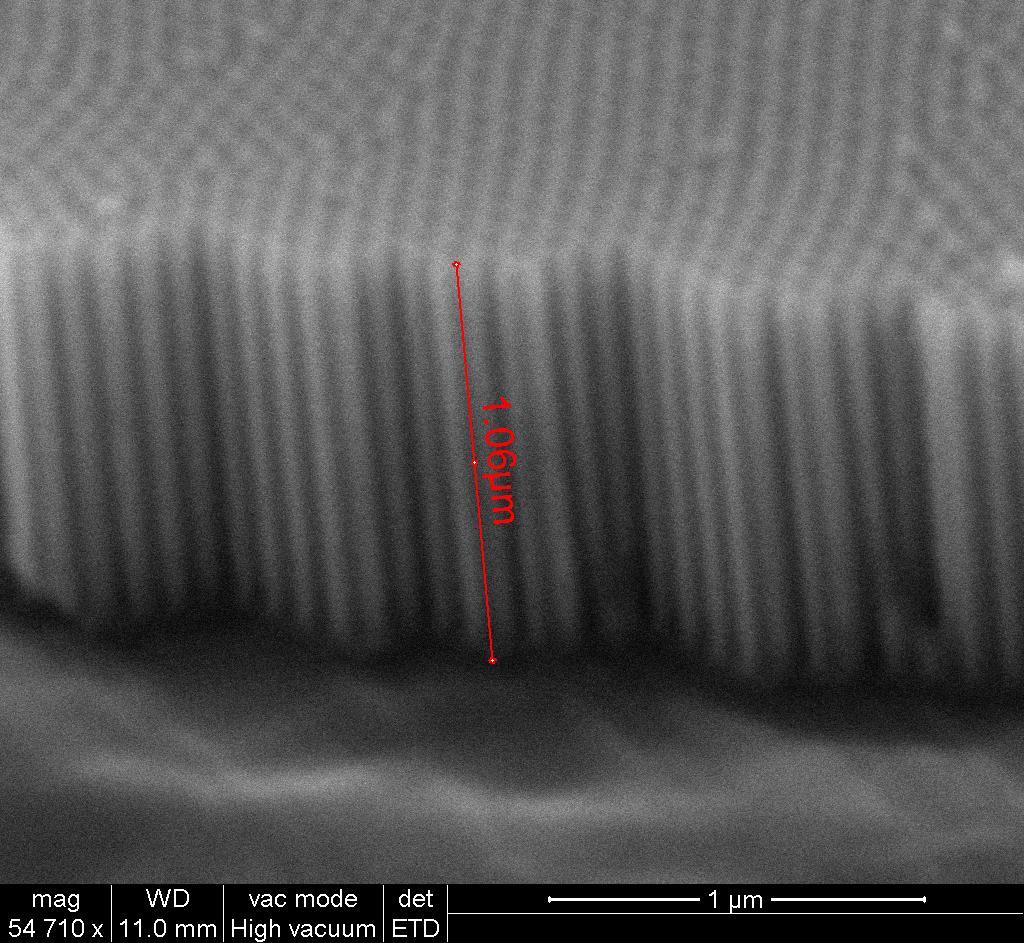


111005-Al1 (40V)

111006-Al1 (50V)
